# Effect of breastfeeding promotion interventions on cost-effectiveness of rotavirus immunization in Indonesia

**DOI:** 10.1186/1471-2458-13-1106

**Published:** 2013-12-01

**Authors:** Auliya A Suwantika, Maarten J Postma

**Affiliations:** 1 Unit of PharmacoEpidemiology & PharmacoEconomics (PE2), Department of Pharmacy, University of Groningen, Groningen, The Netherlands; 2 Faculty of Pharmacy, University of Padjadjaran, Bandung, Indonesia

**Keywords:** Rotavirus, Cost-effectiveness, Vaccination, Breastfeeding, Affordability

## Abstract

**Background:**

Rotavirus infection has been reported to be responsible for the majority of severe diarrhea in children under-5-years-old in Indonesia. Breast milk is considered to give protection against rotavirus infection. Increasing breastfeeding promotion programs could be an alternative target to reduce the incidence of rotavirus diarrhea. This study aims to investigate the effect of breastfeeding promotion interventions on cost-effectiveness of rotavirus immunization in Indonesia, focusing on breastfeeding education and support interventions.

**Methods:**

An age-structured cohort model was developed for the 2011 Indonesia birth cohort. We compared four interventions in scenarios: (i) base-case (***I***_***0***_) reflecting the current situation for the population of under-5-years-old, (ii) with an additional breastfeeding education intervention (***I***_***1***_), (iii) with a support intervention on initiation and duration (***I***_***2***_) and (iv) with both of these two interventions combined (***I***_***3***_). The model applied a 5-years time horizon, with 1 month analytical cycles for children less than 1 year of age and annually thereafter. Monte Carlo simulations were used to examine the economic acceptability and affordability of rotavirus vaccination.

**Results:**

Rotavirus immunization would effectively reduce severe cases of rotavirus during the first 5 years of a child's life even assuming various breastfeeding promotion interventions. The total yearly vaccine cost would amount to US$ 64 million under the market vaccine price. Cost-effectiveness would increase to US$ 153 per quality-adjusted-life-year (societal perspective) with an optimal breastfeeding promotion intervention. Obviously, this is much lower than the 2011 Gross Domestic Product (GDP) per capita of US$ 3,495. Affordability results showed that at the market vaccine price, rotavirus vaccination could be affordable for the Indonesian health system.

**Conclusions:**

Rotavirus immunization would be a highly cost-effective public health intervention for Indonesia even under various breastfeeding promotion interventions based on the WHO’s criteria for cost-effectiveness in universal immunization.

## Background

Despite the common practice of breastfeeding in developing countries, exclusive breastfeeding remains uncommon [1]. There is still a big challenge for healthcare professionals to encourage women to breastfeed exclusively. The World Health Organization (WHO) recommends exclusive breastfeeding for the first 6 months of life and continuing partially breastfeeding up to 2 years of age and beyond. Obviously, a focus both on initiating and continuing breastfeeding is very important to reduce the risk of failure on breastfeeding start and maintenance [2]. Yet, practice deviates from these recommendations. In particular, the low uptake of exclusive breastfeeding might be caused by the lack of breastfeeding support and education [3].

The WHO estimated that optimized breastfeeding would save 1.45 million children’s lives each year in developing countries due to averting diarrhea and respiratory tract infections [4]. In Indonesia, rotavirus infection has been reported to be responsible for the majority of severe diarrhea in children under-5-years-old, mainly in children aged between 6–24 months old [5]. Breast milk is considered to give protection against rotavirus infection because it contains anti-rotavirus maternal antibodies and other nonspecific inhibitors [6]. Therefore, increasing breastfeeding promotion programs in Indonesia could be an alternative target to reduce the incidence of rotavirus diarrhea.

In terms of the health economic perspective, a previous study confirmed that implementation of rotavirus vaccination in Indonesia could be cost-effective [7]. Notably, the previous study did not take breastfeeding explicitly into account and did not consider the effect of potential breastfeeding promotion interventions on cost-effectiveness of rotavirus immunization. Considering these limitations of the previous study and the need to explore the impact of breastfeeding promotion in childhood vaccination, we investigated the effect of breastfeeding promotion interventions on cost-effectiveness of rotavirus immunization in Indonesia, focusing on breastfeeding education and support intervention. Specifically, optimized breastfeeding might impact the economic evaluation results since maternal protection would be enhanced. Yet, cost-effectiveness could still be acceptable even in that situation. In this study we applied a cost-effectiveness model developed by University of Groningen labeled “Consensus Model on Rotavirus Vaccination” (CoRoVa), in the context of the Indonesian healthcare system for the next 5 years [8].

## Methods

**Figure 1:**
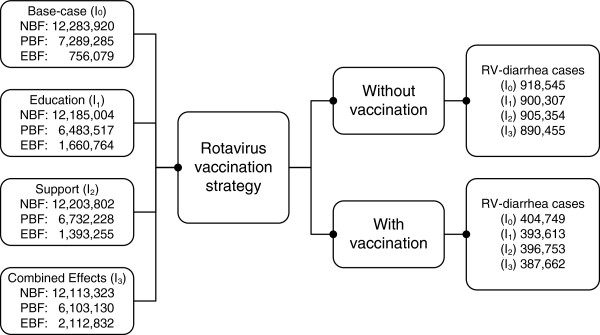
Scheme for estimation of RV-diarrhea cases for all interventions.

We applied the CoRoVa model, previously used to estimate cost-effectiveness of rotavirus vaccination both in developing and developed countries. The model is extensively validated and has the ability to calculate potential impact of breastfeeding on vaccination cost-effectiveness. Considering 4,200,000 infants [7] reflecting 2011 Indonesia birth cohort and using the data from the Indonesian Demographic and Health Survey (IDHS) on the age-specific breastfeeding patterns [9], we constructed an age-structured model with a 5-years time horizon based on breastfeeding statuses. In particular, we considered exclusive, partial or no breastfeeding as categories. In this study, we compared four scenarios: (i) base-case (***I***_***0***_) reflecting the current situation for the population of under-5-years-old, (ii) with an additional breastfeeding education intervention (***I***_***1***_), (iii) with a support intervention on initiation and duration (***I***_***2***_) and (iv) with both of these two interventions combined (***I***_***3***_) (see Figure 1). The 5-year time horizon was chosen as rotavirus infection has been reported to be responsible for the majority of severe diarrhea cases in population under-5-years-old in Indonesia, and the severity rapidly decreases over 5 years [5]. Based on previous studies, each intervention could be assumed to increase the breastfeeding rate and correspondingly reduce the incidence of rotavirus-diarrhea [1,3,10-12]. Aligning with the global health outcomes chosen in the previous study, we used the four severity levels of rotavirus-diarrhea; i.e. mild (home treatment), moderate (general practitioner treatment), severe (hospitalization) and death [13]. We ran the model in Microsoft Excel 2010 and used @ Risk 4.5.4 for probabilistic sensitivity analysis.

Applying the data from the 2007 IDHS on breastfeeding status by age and considering the WHO’s recommendation on breastfeeding duration, we populated the under-5-years-old population in Indonesia into the relevant age groups (0–5, 6–11, 12–23, 24–35, 36–47 and 48–59 months) based on breastfeeding statuses: exclusive breastfeeding (EBF), partial breastfeeding (PBF) and no breastfeeding (NBF) [9,14]. We applied the actual distribution over different breastfeeding modes in under-6-months-old population: 32.4% EBF, 59.1% PBF and 8.5% NBF [9]. For 6–11, 12–23 and 24–35 months, we applied the proportions of EBF:PBF:NBF at 3.1%:82.0%:14.9%, 0.3%:31.7%:68.0% and 0.1%:30.1%:69.8%, respectively [9]. For 36–47 months, the proportions were assumed to be 0%:10%:90%, and for 48–59 months, we assumed 100% NBF.

Due to lack of data on 2011 diarrhea cases for under-5-years-old in Indonesia, we estimated 2011 diarrhea cases over breastfeeding statuses by applying 2007 data on diarrhea cases and considering the WHO’s data on relative risk of diarrhea morbidity by feeding statuses [15]. Additionally, we made an assumption that the same number of diarrhea cases as estimated for 2007 would overall apply to 2011 in Indonesia as well. Based on a 2009 study on economic evaluation of a routine rotavirus vaccination in Indonesia [7], we divided rotavirus-diarrhea cases into respective levels of severity by using proportions of 76.0%, 22.9% and 1.16% for outpatient visits (moderate and mild cases), hospitalization (severe cases) and fatal cases. Additionally, we estimated that moderate cases would be 38.7% from mild cases [16]. These numbers would subsequently reflect diarrhea cases in the base-case (***I***_***0***_) in our study.

Assuming ***I***_***0***_ as condition without breastfeeding promotion interventions in Indonesia, we compared it with three intervention scenarios as mentioned. Firstly, we assumed a breastfeeding education intervention (***I***_***1***_). Based on the WHO’s 2009 study in Iran [10], we assumed the same method and considered the same effect on increasing the rate of EBF. This method was initiated when a mother stayed for 24-hours in a postpartum ward [10]. It required a 40-hours training in total during several days by on the advantages of breastfeeding and the importance of EBF [10]. On the initial day of discharge, the mother and her baby were observed on taking the right breastfeeding position with follow-up visits at days 10, 15 and 30 after delivery [10]. Considering the odds ratio (OR) on feeding patterns after 4 months delivery in the study and control groups between EBF (OR = 16.89; 95%CI = 5.42-52.59; p < 0.0001), PBF (OR = 0.22; 95%CI = 0.10-0.49; p = 0.0002) and NBF (OR = 0.24; 95%CI = 0.06-0.92; p = 0.04) [10], we estimated the EBF-rate could be 120% higher compared to cases without an intervention on a 5-years time horizon. We assumed that the increasing rate of EBF reduces proportions of PBF and NBF at the same rates in each age group; i.e. the relative sizes of PBF and NBF remain the same as in ***I***_***0***_. We estimated the program could reduce rotavirus-diarrhea cases for under-5-years-old by 2% [10,11,17].

Secondly, we analyzed breastfeeding support intervention (***I***_***2***_). This method has been applied among a group of low income women in the US, where they were trained and motivated on initiating and continuing to breastfeed and had to meet a lactation consultant individually to discuss breastfeeding during pregnancy [3]. This method was followed-up at 4 days, 2-3-4-6 weeks, 3-4-5-6 months postpartum to ascertain on managing and continuing to breastfeed [3]. Applying the OR proportion of women breastfeeding after 6 months intervention [3] and considering the mean difference for education (***I***_***1***_) and support (***I***_***2***_) with three outcome measures (initiation, short-term and long-term duration) [18], we estimated the program could increase the proportion of EBF up to 84% higher as compared to no program and could reduce rotavirus-diarrhea cases for under-5-years-old by 1.4% [3,11,15,18].

Thirdly, we assumed a combined effect of education and support interventions (***I***_***3***_) [18]. We considered to apply the best effect between education, support and reported combined effects for initiation (mean difference = 23%; 95%CI = 12%-34%), short-term (mean difference = 23%; 95%CI = 12%-34%) and long-term duration (mean difference = 23%; 95%CI = 12%-34%), respectively [18]. We calculated that the combined effect could increase the probability of EBF up to 179% higher as compared to no program and could reduce rotavirus-diarrhea cases for under-5-years-old by 3% [3,11,15,18]. For all intervention scenarios (***I***_***1***_, ***I***_***2***_ and ***I***_***3***_), we assumed the proportion of rotavirus-diarrhea cases in each age group to remain the same as in ***I***_***0***_.

We assumed that the RotaTeq vaccine in three doses is used in our study. For formula-fed infants (NBF), we applied the rotavirus vaccine efficacies at 70%, 84% and 76.5% as initial vaccine effectiveness for mild-moderate, severe and fatal cases, respectively [7]. We calculated the vaccine efficacies for breastfed infants (EBF and PBF) at 63.1%, 75.7% and 68.9% for mild-moderate, severe and fatal cases, respectively, by considering the results from a study by Vesikari *et al.* on rotavirus vaccine efficacy in breast-fed European infants, and a comparative study on rotavirus vaccine efficacy in low, middle and high socio-economic settings by Lopman *et al.*[6,19]. Based on a previous study, we assumed that the vaccine effectiveness would exponentially decreased by 11% per year [20]. For between-dose efficacy, we applied 82% (between doses 1 and 2) and 84% (between doses 2 and 3) of full effectiveness [20,21]. We applied 2011 DPT vaccine coverage at 94% [22] as rotavirus vaccine coverage. We obtained the quality-adjusted-life-year (QALY) losses data by considering the disutility of each severity level and its duration from earlier works [16]. However, in this study we did not consider QALY-losses in caregivers.

In terms of economic perspectives, we analyzed the data both from the healthcare and societal perspectives. From the healthcare perspective, we only considered direct medical costs while from the societal perspective we considered broader cost items (direct medical, direct non-medical and indirect cost) [7]. We calculated all costs due to rotavirus-diarrhea for severe and moderate cases by considering 2007 data on hospitalization and outpatient visit costs due to rotavirus-diarrhea in Indonesia [7]. For mild cases, we estimated direct medical cost from the expenditure per child of oral-rehydration-therapy (ORT) in diarrhea treatment for children under-5-years-old [23]. We converted these costs into 2011 US$ by considering the annual inflation rates.

Finally, we compared the reduction in rotavirus cases, QALY losses, and the cost-of-illness due to rotavirus-diarrhea. Using a market price of US$ 5 per dose [20], we estimated the incremental cost-effectiveness ratio (ICER) per QALY for all four scenarios. Applying the WHO’s definition on cost-effectiveness of universal immunization according to the GDP-per-capita, (i) highly cost-effective (less than one GDP-per-capita); (ii) cost-effective (between 1 and 3 times GDP-per-capita); and (iii) cost-ineffective (more than 3 times GDP-per-capita) [24], we specifically evaluated the results of rotavirus vaccination in Indonesia in all four scenarios.

We performed univariate and probabilistic sensitivity analyses. We investigated the effects of different input parameters by varying each parameter at value of ± 25% while keeping other parameters constant in univariate sensitivity analyses. In the context of breastfeeding promotion interventions in this study, we varied from the minimal intervention (***I***_***2***_) to the maximal intervention (***I***_***3***_). Furthermore, probabilistic sensitivity analysis (PSA) was performed by running 5000 Monte Carlo simulations. We evaluated the affordability related to the required budget for vaccination (vaccination costs + treatment costs) to analyze the budget impacts on the implementation of vaccination from the healthcare perspective, which is relevant for assisting decision makers in the health sector. All input parameters are shown in Table 1.

**Table 1:** Parameters used in the economic model

**Parameters**	**Base-case value**	**Distribution**	**References**
Vaccine coverage	94%	Triangular (89%; 94%; 99%)	[22]
Vaccine efficacy in formula-fed infants			
Mild	70.0%	Triangular (66.5%; 70.0%; 73.5%)	[7]
Moderate	70.0%	Triangular (66.5%; 70.0%; 73.5%)	
Severe	84.0%	Triangular (79.8%; 84.0%; 88.2%)	
Death	76.5%	Triangular (72.7%; 76.5%; 80.3%)	
Vaccine efficacy in breast-fed infants			
Mild	63.1%	Triangular (59.9%; 63.1%; 66.2%)	[6,7,9]; calculated
Moderate	63.1%	Triangular (59.9%; 63.1%; 66.2%)	
Severe	75.7%	Triangular (71.9%; 75.7%; 79.5%)	
Death	68.9%	Triangular (65.5%; 68.9%; 72.4%)	
Rotavirus-diarrhea cases			
Base-case			
EBF	15,626	Normalised mean: 15,626 (90%CI; 15,382-15,871)	[7,9,14-16,20]; calculated
PBF	452,658	Normalised mean: 452,658 (90%CI; 451,412-453,903)	
NBF	450,261	Normalised mean: 450,261 (90%CI; 449,018-451,504)	
Education			
EBF	36,674	Normalised mean: 36,674 (90%CI; 36,300-37,047)	[7,9,10,14-16,20]; calculated
PBF	420,264	Normalised mean: 420,264 (90%CI; 419,058-421,469)	
NBF	442,056	Normalised mean: 442,056 (90%CI; 440,824-443,289)	
Support			
EBF	38,321	Normalised mean: 38,321 (90%CI; 37,940-38,703)	[3,7,9,14-16,18,20]; calculated
PBF	422,586	Normalised mean: 422,586 (90%CI; 421,378-423,795)	
NBF	441,548	Normalised mean: 441,548 (90%CI; 440,316-442,780)	
Combined Effects			[7,9,14-16,18,20]; calculated
EBF	57,824	Normalised mean: 57,824 (90%CI; 57,356-58,292)	
PBF	394,543	Normalised mean: 394,543 (90%CI; 393,371-395,715)	
NBF	433,441	Normalised mean: 433,441 (90%CI; 432,220-434,663)	
Utility losses			
Mild	0.00164	Triangular (using 25% lower and upper)	[16,20]
Moderate	0.00548		
Severe	0.02110		
Death	1.00000		
Total medical direct costs per case (healthcare perspective, US$)			
Mild	1.08	Triangular (1.08; 1.44; 1.80)	[23]
Moderate	4.31	Triangular (3.23; 4.31; 5.39)	[7]
Severe	41.72	Triangular (31.29; 41.72; 52.15)	[7]
Total direct and indirect costs per case (societal perspective, US$)			
Mild	2.81	Triangular (2.11; 2.81; 3.52)	[23]
Moderate	5.69	Triangular (4.27; 5.69; 7.11)	[7]
Severe	56.34	Triangular (42.25; 56.34; 70.42)	[7]
Total vaccination and administration cost (per child, US$)			
3-dose, Market price	15.50	Alternative scenario	[7,20]
Discount rate	3%	Unvaried	[14,20]

## Results

Assuming 94% vaccine coverage [22], vaccination of 4,200,000 birth cohort [7] would reduce rotavirus-diarrhea with 513,796 cases in ***I***_***0***_. Considering breastfeeding promotion interventions, vaccination would reduce rotavirus-diarrhea by 506,694; 508,601 and 502,793 for ***I***_***1***_, ***I***_***2***_ and ***I***_***3***_, respectively (see Table 2). From the societal perspective, vaccination would save US$ 8,369,236 in cost-of-illness due to rotavirus-diarrhea in ***I***_***0***_ and with breastfeeding promotion interventions it would save US$ 8,245,065; US$ 8,277,719 and US$ 8,177,772 for ***I***_***1***_, ***I***_***2***_ and ***I***_***3***_, respectively. For QALYs loss, vaccination would save 348,887 discounted QALYs in ***I***_***0***_ and with breastfeeding promotion interventions it would save 343,534; 344,926 and 340,653 for ***I***_***1***_, ***I***_***2***_ and ***I***_***3***_, respectively.

**Table 2:** Results for all interventions

	**No Vaccination**	**Vaccination **^**j**^	**Difference**
**Base-case (I**_**0**_**) **^**a**^			
Number of RV-diarrhea cases ^e,f^	918,545	404,749	513,796
Mild cases	503,190	243,800	259,390
Moderate cases	194,734	76,214	118,520
Severe cases	209,970	80,084	129,886
Death cases	10,651	4,651	6,000
QALYs lost ^g^	616,904	268,027	348,887
Cost of illness (healthcare perspective) ^g,h^	$ 9,887,025	$ 3,839,484	$ 6,047,541
Cost of illness (societal perspective) ^g,i^	$ 13,748,048	$ 5,378,812	$ 8,369,236
**Education (I**_**1**_**) **^**b**^			
Number of RV-diarrhea cases ^e,f^	900,307	393,613	506,694
Mild cases	493,198	237,539	255,659
Moderate cases	190,868	73,765	117,103
Severe cases	205,801	77,784	128,017
Death cases	10,440	4,525	5,915
QALYs lost ^g^	603,799	260,265	343,534
Cost of illness (healthcare perspective) ^g,h^	$ 9,681,360	$ 3,723,490	$ 5,957,870
Cost of illness (societal perspective) ^g,i^	$ 13,462,069	$ 5,217,004	$ 8,245,065
**Support (I**_**2**_**) **^**c**^			
Number of RV-diarrhea cases ^e,f^	905,354	396,753	508,601
Mild cases	495,963	239,315	256,648
Moderate cases	191,938	74,435	117,503
Severe cases	206,955	78,442	128,513
Death cases	10,498	4,561	5,937
QALYs lost ^g^	607,368	262,442	344,926
Cost of illness (healthcare perspective) ^g,h^	$ 9,737,646	$ 3,756,191	$ 5,981,455
Cost of illness (societal perspective) ^g,i^	$ 13,540,335	$ 5,262,616	$ 8,277,719
**Combined Effects (I**_**3**_**) **^**d**^			
Number of RV-diarrhea cases ^e,f^	890,455	387,662	502,793
Mild cases	487,802	234,175	253,627
Moderate cases	188,779	72,480	116,309
Severe cases	203,549	76,549	127,000
Death cases	10,325	4,458	5,867
QALYs lost ^g^	596,795	256,142	340,653
Cost of illness (healthcare perspective) ^g,h^	$ 9,571,093	$ 3,661,825	$ 5,909,268
Cost of illness (societal perspective) ^g,i^	$ 13,308,742	$ 5,130,971	$ 8,177,772

^a^ Actual distribution over the different BF patterns.

^b^ Population <2 years old with 100% EBF.

^c^ Population <2 years old with 100% PBF.

^d^ Population <2 years old with 100% NBF.

^e^ Population <5 years old.

^f^ Undiscounted.

^g^ Discounted.

^h^ Direct medical cost.

^i^ Direct medical + direct non-medical + indirect cost.

^j^ Costs are excluding vaccination cost.

**Figure 2:**
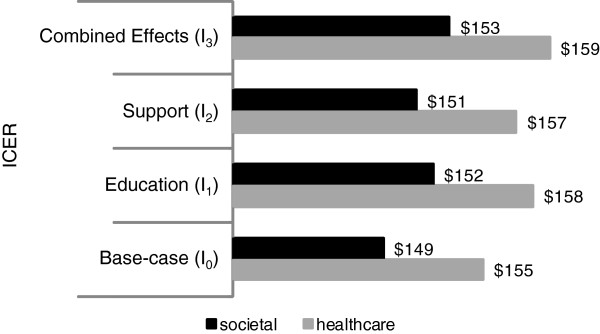
Cost-effectiveness value for all interventions.

With a market price of US$ 5 per dose [20], cost-effectiveness values from the societal perspective are US$ 149, US$ 152, US$ 151 and US$ 153 for ***I***_***0***_, ***I***_***1***_, ***I***_***2***_ and ***I***_***3***_, respectively (see Figure 2). The results are far below the 2011 Indonesian GDP-per-capita of US$ 3,495 [25]. Obviously, according to the WHO’s definition for cost-effectiveness [24], rotavirus immunization is a highly cost-effective intervention even assuming various breastfeeding promotion interventions. Notably, with an optimal breastfeeding promotion intervention (***I***_***3***_), cost-effectiveness would increase to US$ 153 (societal perspective), although the cost-effectiveness is still far below the WHO threshold.

**Figure 3:**
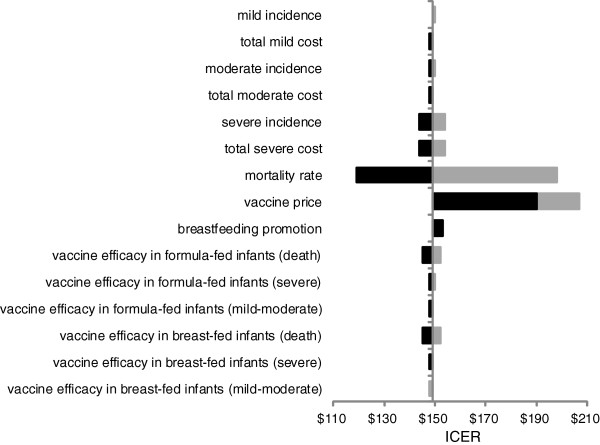
Results of univariate sensitivity analyses.

The impacts of parameter changes on the ICERs are shown in a tornado chart (see Figure 3). The results confirmed that the mortality rate and vaccine price were the most influential parameters in the sensitivity analyses. The cost-effectiveness results were not sensitive to the mild cost, moderate cost, severe cost, mild incidence, moderate incidence, severe incidence, breastfeeding promotions and vaccine efficacies.

**Figure 4:**
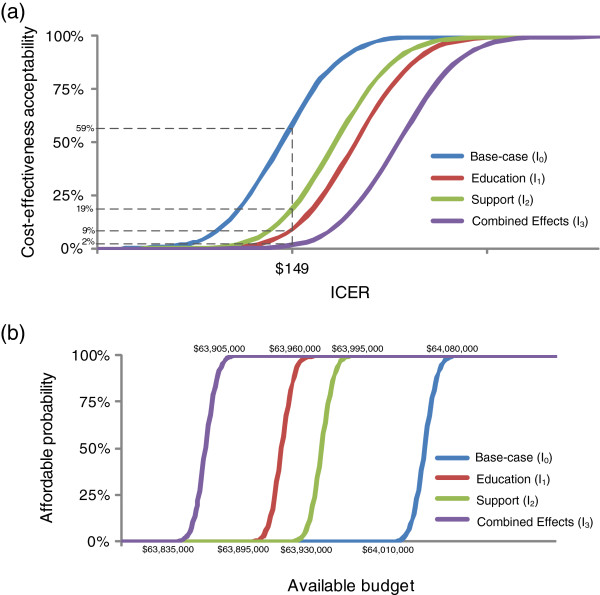
**Cost-effectiveness acceptability and affordability curves. (a)** Cost-effectiveness acceptability curves from the societal perspective. **(b)** Affordability curves from the healthcare perspective.

At a threshold ICER of US$ 149 (the base case value from the societal perspective), the probability for the vaccination program to be cost-effective would be 59%; 9%; 19% and 2% for ***I***_***0***_, ***I***_***1***_, ***I***_***2***_ and ***I***_***3,***_ respectively. Already at a threshold ICER of US$ 158, the probability for the vaccination program to be cost-effective would be 100% for all scenarios (see Figure 4a). From the healthcare perspective, rotavirus immunization with a market price of US$ 5 per dose would always be implementable when the budget exceeds US$ 64,080,000; US$ 63,960,000; US$ 63,995,000 and US$ 63,905,000 for ***I***_***0***_, ***I***_***1***_, ***I***_***2***_ and ***I***_***3***_, respectively (see Figure 4b).

## Discussion

Compared to the base case (***I***_***0***_), breastfeeding interventions would increase EBF-rate by 120%, 84% and 179% for ***I***_***1***_, ***I***_***2***_ and ***I***_***3***_, respectively, as mentioned. At the market vaccine price of US$ 5 per dose, a rotavirus immunization program in Indonesia could reduce rotavirus-diarrhea by 259,390; 118,520; 129,886 and 6,000 for mild, moderate, severe and fatal cases, respectively. Assuming the base-case (***I***_***0***_) as a condition without breastfeeding promotion interventions in Indonesia, breastfeeding promotion interventions could reduce rotavirus-diarrhea from ***I***_***0***_ by 1.4% - 3.1% for ***I***_***1***_, ***I***_***2***_ and ***I***_***3***_. From the social perspective, the incremental cost-effectiveness ratios are US$ 149; US$ 152; US$ 151 and US$ 153 for ***I***_***0***_, ***I***_***1***_, ***I***_***2***_ and ***I***_***3***_, respectively. Notably, our assumption that breastfeeding might decrease the effectiveness of rotavirus immunization is congruent with other studies [4,6,26]. Furthermore, based on the WHO’s criteria for cost-effectiveness in universal vaccination, our results confirm that rotavirus vaccination would be a highly cost-effective public health intervention for Indonesia even under various breastfeeding promotion interventions [24].

In our study we took uncertainties into account by using univariate and probabilistic sensitivity analyses. The sensitivity analyses showed that the mortality rate and vaccine price were the most influential parameters impacting the cost-effectiveness results. The results on this study reconfirmed the results from previous studies on cost-effectiveness of rotavirus immunization [16,27]. Postma *et al.* also found that the cost-effectiveness of rotavirus immunization in South East Asia Region (SEAR) was highly sensitive to the mortality rate and vaccine price [16]. A critical review on cost-effectiveness of rotavirus vaccination previously also mentioned mortality as the most influential parameter for middle and low income countries [27].

We found a similar required funds (vaccination and treatment costs) for childhood vaccination. Considering the market vaccine price of US$ 5 per dose, rotavirus vaccination would require a budget of US$ 63,905,000 – US$ 64,080,000. Despite all interventions required the same budget for the vaccination costs, the results indicated that breastfeeding promotion could provide some potentials to decrease the treatment costs for rotavirus immunization in Indonesia due to its effect on reducing rotavirus-diarrhea cases. Thus, breastfeeding promotion interventions could potentially result in less opportunities for cost offsets of vaccination. Compared to the total Indonesian government health budget for the whole immunization program in 2011 (US$ 198 million) [28], the required fund by the government for universal rotavirus vaccination would yet be unrealistic. Next to potentially manufacturing a rotavirus vaccine nationally at much lower costs prices, support could be considered to achieve subsidized affordable prices.

Our study is not the first study on the economic analysis of rotavirus vaccination in Indonesia but it could provide crucial information for the policy makers specifically on the potential introduction of breastfeeding promotion combined with the implementation rotavirus immunization in National Immunization Programmes (NIP). This combined approach would increase the EBF-rate and reduce rotavirus-diarrhea cases at potential cost-effective efforts. We extended the CoRoVa model that has been used in a previous study to calculate cost-effectiveness of rotavirus for both developed and developing countries, by taking breastfeeding into account and investigating explicitly the effect of breastfeeding promotion interventions on cost-effectiveness of rotavirus immunization in Indonesia. The relationships between breastfeeding practice, breastfeeding promotion and rotavirus-diarrhea are well-known and therefore important to be included in our modeling approach. Despite that several other interventions on breastfeeding promotion were mentioned in previous study [29], in this study we limited ourselves to two mostly-used interventions of breastfeeding education and support intervention.

We noted several limitations in our study. Firstly, due to lack of data on herd immunity, we had to apply a static model instead of a dynamic model. If we were to include herd immunity in a dynamic analysis there would be greater impacts of rotavirus vaccination and its cost-effectiveness would be further improved. Secondly, we noted lack of 2011 data on rotavirus-diarrhea incidence in Indonesia and actual data on implementation of breastfeeding promotion interventions. We applied 2007 data on rotavirus-diarrhea incidence from a previous study and assumed it would be the same with 2011, while for the implementation of breastfeeding promotion interventions, we assumed that the base-case is a condition without specific breastfeeding promotion interventions in recent years. To overcome this limitation, we varied these assumptions extensively in multiple sensitivity analysis. Finally, we only obtained treatment costs for 2007 and we adjusted to 2011 values by considering the inflation rate.

Due to the low uptake of EBF in Indonesia and the high prevalence of rotavirus-diarrhea in population under-5-years-old, policy makers in Indonesia should consider taking action to enhance the introduction of rotavirus vaccination and designing more intensive programs on breastfeeding promotion interventions. We showed that this approach is potentially highly cost-effective. However, as a developing country, Indonesia is faced with limited resources especially on providing required budget both for implementation of rotavirus vaccination and breastfeeding promotion interventions. Getting funds from international organizations could be realistic sollution to overcome this problem. Hopefully, this assists the Indonesian government in designing optimal policies both in increasing the EBF-rate and in reducing the incidence of rotavirus-diarrhea.

## Conclusion

Rotavirus immunization is a highly cost-effective intervention for the Indonesian healthcare system even under various breastfeeding promotion interventions based on the WHO’s criteria for cost-effectiveness in universal immunization. Yet, the implementation of rotavirus immunization in Indonesia would be unrealistic without international organization support.

## Competing of interests

MJP received grants, honoraria and travel stipends from various pharmaceutical companies, inclusive those interested in the subject matter of this paper. AAS has no relevant affiliations or financial involvement with any organizations or entity with a financial interest in or financial conflict with the subject matter or material discussed in the manuscript.

## Authors’ contributions

AAS conceived and designed this study, collected and analyzed the data, and drafted the manuscript. MJP contributed to the drafting and critical revision of the manuscript. All of the authors read and approved the final manuscript.

## Pre-publication history

The pre-publication history for this paper can be accessed here:

http://www.biomedcentral.com/1471-2458/13/1106/prepub
